# Targeting miR-146a to Treat Delayed Wound Healing in Human Diabetic Organ-Cultured Corneas

**DOI:** 10.1371/journal.pone.0114692

**Published:** 2014-12-09

**Authors:** Michael A. Winkler, Christian Dib, Alexander V. Ljubimov, Mehrnoosh Saghizadeh

**Affiliations:** 1 Eye Program, Board of Governors Regenerative Medicine Institute, Departments of Biomedical Sciences, Cedars-Sinai Medical Center, Los Angeles, California, United States of America; 2 Department of Neurosurgery, Cedars-Sinai Medical Center, Los Angeles, California, United States of America; 3 David Geffen School of Medicine at University of California Los Angeles, Los Angeles, California, United States of America; Wayne State University, United States of America

## Abstract

Limbal epithelial stem cells (LESC) residing at the corneal periphery are largely responsible for maintaining corneal optical transparency by continuously supplying new corneal epithelial cells, which mature during their radial migration to the central cornea. Diabetes mellitus (DM) affects all the structures of the eye including the cornea. Frequent epithelial erosions, delayed wound healing, and microbial infections are common alterations of the diabetic eye that can result in vision loss. MicroRNAs (miRNAs) are short non-coding oligonucleotides that regulate gene expression by repressing translation. Our purpose was to understand the role of miR-146a in the human limbal versus central corneal epithelial compartment in normal and pathological conditions such as diabetes mellitus. Using quantitative real-time PCR (QPCR) we found miR-146a enrichment in the limbal corneal compartment. This miRNA was also expressed at higher levels in the diabetic vs. normal limbus. Cell migration and wound closure were significantly delayed in normal and diabetic primary limbal epithelial cells (LEC) transfected with miR-146a. Cells treated with miR-146a had decreased levels of phosphorylated (activated) p38 and EGFR, mediators of epithelial wound healing. Conversely, inhibition of miR-146a significantly enhanced cell migration in both normal and diabetic primary LEC and in diabetic organ-cultured corneas by nearly 40% vs. scrambled miRNA control, accompanied by increased phosphorylated signaling intermediates. Transfection of miR-146a in cultured LEC resulted in an increased immunoreactivity for putative LEC markers Frizzled-7 and K15, whereas inhibition of miR-146a decreased their expressions. These data suggest that miR-146a plays a role in LEC maintenance at the corneal periphery, and its expression is downregulated during their migration towards the central cornea and accompanying terminal differentiation. Furthermore, abnormal miR-146a upregulation may be an important mechanism of delayed wound healing in the diabetic cornea.

## Introduction

Diabetes mellitus (DM) is a metabolic disease currently affecting nearly 29 million people in the United States alone [1]. This number is projected to increase with the expanding population. Chronic DM-associated complications include renal failure, limb neuropathies, cardiovascular problems, and vision loss. Clinically, DM elicits many deleterious pathologic changes that affect different parts of the eye including the cornea [2]–[5].

Corneal epithelium is constantly renewed by limbal epithelial stem cells (LESC) located at the corneal periphery [6]–[8]. In normal corneal homeostasis LESC give rise to progeny, transient amplifying (TA) cells, which differentiate into mature corneal epithelium during their radial migration towards the central cornea [9]. The consistent generation of new corneal epithelium provides a means for maintaining a transparent cornea, which is required for optimal visual clarity. In addition, the corneal epithelium provides a barrier against microbial and noxious agents. In cases of aberrant LESC numbers or function, normal visual acuity can be affected. We have previously shown that diabetic corneas have significantly decreased expression of several LESC markers, which could contribute to their dysfunction and lead to diabetic keratopathy [10], [11].

MicroRNAs (miRNA) are short non-coding oligonucleotides (18–22 nt) that regulate gene expression at the translational level either by binding the 3′-UTR of messenger RNA and loading it onto the RISC complex for degradation, or by physically inhibiting mRNA passage through the ribosome [12]–[14]. Recent work by numerous groups has documented the significance of miRNA in the regulation of many cellular processes including differentiation, proliferation, migration, and apoptosis [15]–[19]. Whereas some studies have shown the location and function of microRNA in the mouse eye [20], little progress has been made in understanding the role of miRNA in human corneal homeostasis [21]–[25].

Previously, we uncovered the repertoire of miRNA expressed in the central compartment of normal and diabetic corneas using microarray analysis [25]. In the present study we used gain-of-function and loss-of-function strategies in human primary limbal epithelial cells and organ-cultured corneas to demonstrate the role of miR-146a in LESC homeostasis and wound healing.

## Materials and Methods

### Human Corneal Procurement

A total of 11 normal and 14 diabetic age-matched human cadaver corneas were received from National Disease Research Interchange (NDRI; Philadelphia, PA) in Optisol storage medium (Chiron Vision, Claremont, CA) within 48 hours of donor death (Table 1). NDRI uses a human tissue collection protocol approved by a managerial committee and is subject to oversight by National Institutes of Health. This work was covered by IRB protocols EX-1055 and Pro00019393 from Cedars- Sinai Medical Center.

**Table 1:** List of Corneas Used in this Study.

Case Number	Age	Gender	Cause of Death	DM Duration (Years)
N 10–21	79	M	Cerebrovascular Accident	N/A
N 10–22	75	F	Coronary Artery Disease	N/A
N 10–23	57	M	Cerebrovascular Accident	N/A
N 10–24	66	M	Cardiopulmonary Accident	N/A
N 11–11	62	F	Congestive Heart Failure	N/A
N 11–27	34	F	Unknown	N/A
N 13–19	25	M	Multi-trauma	N/A
N 13–21	20	M	Asphyxiation	N/A
N 13–33	79	M	Intracranial Hemorrhage	N/A
N 13–34	79	F	Sequela to Hip Fracture	N/A
N 14–39	78	M	Intracerebral Hemorrhage	N/A
DM 11–18	70	F	Cerebrovascular Accident	20
DM 11–30	39	M	Renal Failure	Unknown
DM 12-2	74	F	Renal Failure	10–15
DM 12-5	51	M	Ventricular Fibrillation	Unknown
DM 13-04	67	M	Coronary Artery Disease	13
DM 13–24	85	F	Anoxic Brain Injury	20
DM 13–27	62	F	Heart Disease	20
DM 13–32	70	M	subdural Hematoma	8
DM 13–35	79	F	Subdural Hematoma	10
DM 13–38	88	M	Respiratory Failure	10
DM 13–40	54	F	Intracranial Hemorrhage	10
DM 14–06	72	M	Renal Failure	Unknown
DM 14–08	50	M	Cardiac Arrest	12
DM 14–23	84	F	Congestive Heart Failure	10

N, normal; DM, diabetic mellitus; DR, diabetic retinopathy; M, male; F, female.

### Isolation of Total RNA

Total RNA including low molecular weight (LMW) RNA was extracted from homogenized autopsy human normal (n = 6) and diabetic (n = 5) 8.5 mm central corneal buttons and adjacent limbal rims using Ambion's mirVana miRNA Isolation Kit (Thermo Fisher Scientific, Waltham, MA) according to the manufacturer's instructions. RNA quality was assessed using a NanoDrop ND-1000 spectrophotometer, a Qubit 2.0 fluorometer (Thermo Fisher Scientific) and Agilent 2100 system (Agilent Technologies, Santa Clara, CA).

### Quantitative Real-Time PCR (QPCR)

QPCR was performed as described previously [25]. Briefly, 10 ng of total RNA were reverse transcribed (RT) using Taqman MicroRNA RT kits and miRNA sequence-specific primers (Thermo Fisher Scientific). QPCR was carried out in MicroAmp Optical 384-well plates using Taqman 2X universal PCR Master Mix (no AmpErase UNG) along with Taqman 20X MicroRNA Assays (Thermo Fisher Scientific). Each well contained 1.33 µl of RT reaction product, 1X Taqman PCR Master Mix, and 1X specific miRNA primer, designed to detect mature miRNAs. Amplification was carried out on the ViiA 7 Real Time PCR System (Thermo Fisher Scientific). Each sample was run in triplicate. Signals were normalized to the U75 housekeeping miRNA run in parallel. A comparative threshold cycle (Ct) method (ΔΔCt) was used to calculate relative miRNA expression.

### Primary Limbal Epithelial Cell Isolation and Maintenance

To generate primary corneal limbal epithelial cells (LEC), corneal endothelium was removed from intact human corneas by gentle rubbing with a cotton swab. Eight and a half mm central corneal buttons were then removed with a trephine and the remaining epithelial cells from corneoscleral rims were isolated by Dispase/Trypsin digestion as previously described [25]–[27]. LEC were grown on a mixture of the human basement membrane proteins (FCL) fibronectin (2 µg/cm^2^; BD Biosciences, San Jose, CA), collagen IV (0.6 µg/cm^2^) and laminin [0.2 µg/cm^2^ (Sigma Aldrich, St. Louis, MO) [28]. LEC were maintained in Epilife containing Human Keratinocyte Growth Supplement (HKGS), N-2 Supplement, B27 Supplement, 1X antibiotic/antimycotic mixture, plus 10 ng/mL epidermal growth factor (EGF) (Thermo Fisher Scientific) and 10 ng/mL keratinocyte growth factor (R&D Systems, Minneapolis, MN) In addition, primary LEC were maintained in 10 µM Rho-associated protein kinase (ROCK) inhibitor (Stemgent, Cambridge, MA) until one medium change prior to passage for experiment.

### Transfection of Human Primary Corneal Epithelial Cells and Organ-Cultured Corneas

Corneal organ cultures were established as described [29] and were maintained in Dulbecco's Modified Eagle's Medium (Thermo Fisher Scientific) with 1X insulin-transferrin-selenite (Sigma-Aldrich), 1X non-essential amino acids, and 1X antibiotic/antimycotic mix (Thermo Fisher Scientific). Eight pairs of diabetic organ-cultured corneas were used. Each cornea in a pair was transfected with either 20–30 nm hsa-miR-146a Pre-miR precursor (miR-146a mimic) or Anti-miR (mir-146a inhibitor, Thermo Fisher Scientific), while the fellow cornea was transfected with control Cy3-labeled scrambled sequence miRNA for 48 hours using Lipofectamine RNAiMAX transfection reagent (Thermo Fisher Scientific). After an additional incubation period of 2–5 days, the transfected corneas were either wounded (see wound healing assay below), or used as non-wounded controls. Both groups were processed for immunohistochemistry, QPCR, or western blotting. Increased expression of miR-146a after transfection with hsa-miR-146a Pre-miR precursor and decreased expression of miR-146a after transfection with Anti-miR were confirmed by QPCR.

### Immunostaining

Cultured primary LEC or 5-µm thick transverse corneal cryostat sections were fixed in either cold 4% paraformaldehyde (PFA) for 15 min at room temperature, 1% formalin for 5 min at room temperature, or 100% methanol at −20°C for 10 min, then washed 3 times for 15 min each with PBS at room temperature. Cells or tissues fixed in PFA were permeabilized in 0.2% Triton X-100 for 10 min at room temperature, washed twice with PBS for 10 min each at room temperature, then blocked for 1 h in a 2% BSA PBS solution at room temperature in a humidified chamber. The slides were incubated with primary antibodies (Table 2) in blocking solution overnight at 4°C. Primary antibodies were then washed off three times with PBS for 15 min each at room temperature followed by a 1 hr incubation of cross-species adsorbed secondary antibodies conjugated with either fluorescein isothicyanate (FITC) or tetramethylrhodamine (TRITC) in the dark at room temperature (Jackson ImmunoReseach Laboratories, West Grove, PA) The secondary antibodies were washed off three times with PBS at room temperature for 15 min each. Slides were mounted in media containing 1.5 µg/mL 4′,6-diamidino-2-phenylindole (DAPI; Vector Labs, Burlingame, CA). For each marker the same exposure time was used when photographing stained sections, and assessment was done by more than one observer. The pictures are representative of two to three independent experiments. Negative controls without a primary antibody were included in each experiment.

**Table 2:** Primary Antibodies Used in this Study.

Primary Antibody	Species	Manufacturer/Part Number	Dilution	Application
β-Actin	Mouse	Sigma-Aldrich/A5316	1∶7000	WB
β-Tubulin	Mouse	Sigma-Aldrich/T4026	1∶400	WB
p-EGFR	Rabbit	Thermo Fisher/44784G	1∶200	WB
p-Akt	Rabbit	Cell Signaling/9271	1∶200	IHC, WB
p-ERK1/2	Rabbit	Cell Signaling/4370	1∶400	WB
p-p38	Rabbit	Cell Signaling/ab38238	1∶20, 1∶200	IHC, WB
Integrin α3β1 (VLA-3)	Mouse	Millipore/MAB1992	1∶5	IHC
Keratin 15	Mouse	Santa Cruz Biotechnology/sc-47697	1∶20, 1∶200	IF, WB
Frizzled-7	Mouse	Millipore/MAB1981	1∶200	IHC

### Wound Healing Assay

**Cultured cells**: Confluent transfected cells were “scratch wounded” with a P1000 pipette tip as described [25]. Wound closure was monitored by phase contrast microscopy. Images were taken at regular intervals over 24 hrs and analyzed with ImageJ software. Average wound area relative to the initial wound area (0 hr) was determined in three independent triplicate assays and was compared to control cells transfected with Cy3-labeled scrambled miRNA (negative control).

**Organ-cultured corneas**: Donor human diabetic corneas from NDRI were organ-cultured and assayed for wound healing and marker expression as published [30], [31]. One cornea from each donor pair was transfected with 30 nM miR-146a inhibitor, whereas the fellow cornea received an equal amount of Cy3-labeled scrambled miRNA control for 48 hr, using RNAiMax. Following transfection, either a 5 mm or 8.5 mm wound (to examine central and limbal epithelial function, respectively) was created using a disk soaked in n-heptanol. Wound healing was assessed daily via light microscope [30], [31].

### Cell Proliferation (MTS) Assay

CellTiter 96 AQ_ueous_ Non-Radioactive Cell Proliferation (MTS) Assay (Promega, Madison, WI) with minor modification to the manufacturer's instructions. Transfected primary limbal cells were seeded in triplicate at 40,000–60,000 cells/well in BM protein coated 48 well plates. Absorbance was read on day 3 and day 7 post-transfection using the DTX 880 (Beckman Coulter, Brea, CA) at 450 nm following 2-hour incubation at 37°C and 5% CO_2_ with MTS reagent. Data were collected with Soft Max Pro 6.3 (Molecular Devices, Sunnyvale, CA). Average numbers of viable cells were calculated using linear regression generated from a standard curve possessing an R-squared value of 0.985 or greater.

### Western Blot Analysis

Western blotting was performed as described previously [25] with some modifications. Briefly, 8% to 16% gradient Tris-glycine SDS polyacrylamide gels were used (Thermo Fisher Scientific). Gel loading was normalized using antibodies AC-74 to β-actin or TUB 2.1 to β-tubulin (Sigma-Aldrich). After transfer to nitrocellulose membranes, blots were blocked in Blotto Blocker (Thermo Fisher Scientific) and incubated with primary antibodies, rabbit anti-p-EGFR (44–784 G, Thermo Fisher Scientific), mouse anti-p-p38 (ab50012, Abcam, Cambridge, MA), rabbit anti-p-ERK1/2 (4370, Cell Signaling) or rabbit anti-pAkt (9271, Cell Signaling). IRDye 800 CW or 680 RD goat anti-mouse or anti-rabbit (Li-Cor Biosciences, Lincoln, NE) was used as secondary antibodies. The blots were scanned using Odyssey CLX imaging system (Li-Cor Biosciences).

### Statistical analysis

Experiments were analyzed by Student's t-test for two groups, or ANOVA for three or more groups with p<0.05 considered significant, using Prism6 (GraphPad Software, San Diego, CA).

## Results

### MiR-146a is differentially expressed in the limbus of normal and diabetic corneas

**Figure 1:**
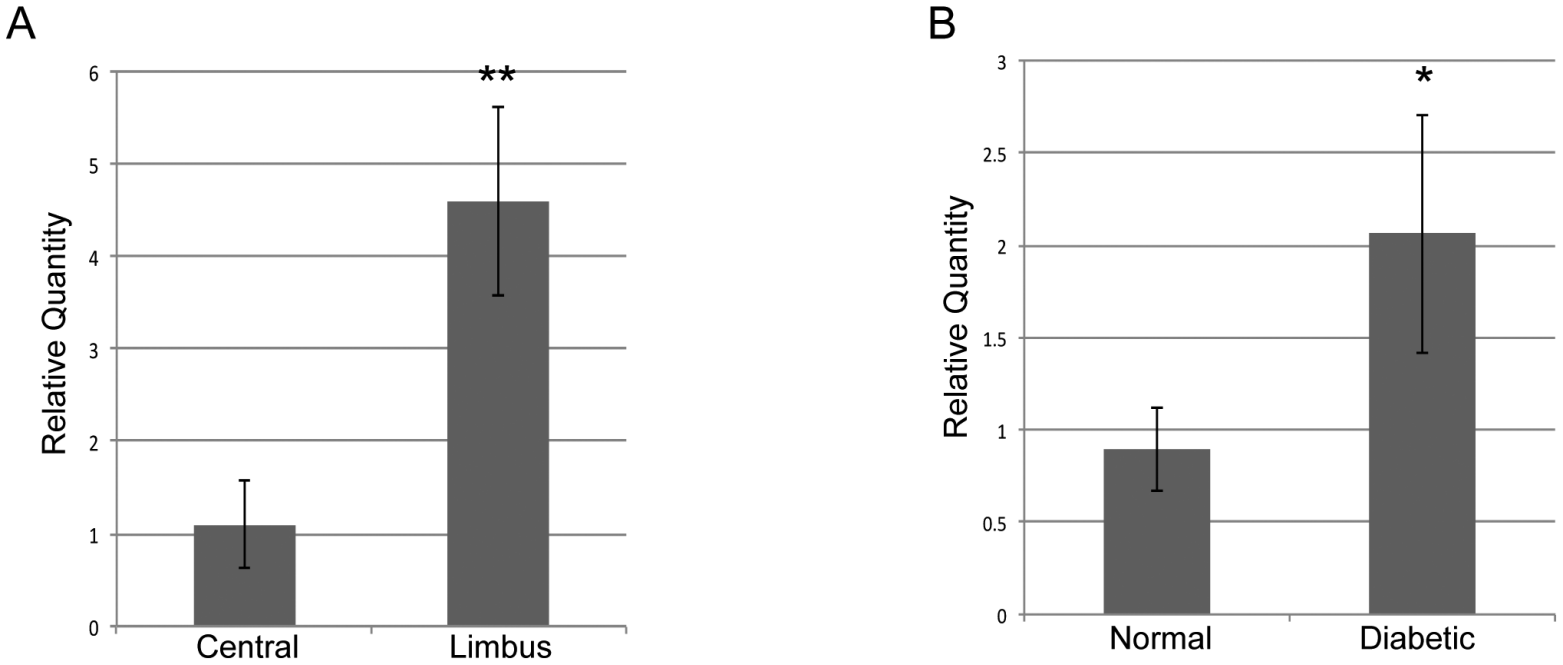
Q-PCR validation of differentially expressed miR-146a. Validation of differentially expressed miR-146a in *ex vivo* central cornea vs. limbus, **A**; and in normal vs diabetic limbus, **B**; by Q-PCR. Bars represent SEM of pooled values (n = 5) ** p<0.001 and * p<0.05 by paired two-tailed t test.

Previously, we have shown miR-146a upregulation in the diabetic central cornea [25]. In the present study, using total RNA from six normal and five diabetic human limbi and central corneas, we demonstrated that in addition, miR-146a was also upregulated in the limbal compartments of the diabetic vs. normal cornea (Fig. 1A). MiR-146a was also overexpressed in limbus vs. central part of both normal and diabetic corneas (Fig. 1B).

### Upregulated miR-146a in diabetic cornea delays epithelial wound healing in LEC *in vitro*

**Figure 2:**
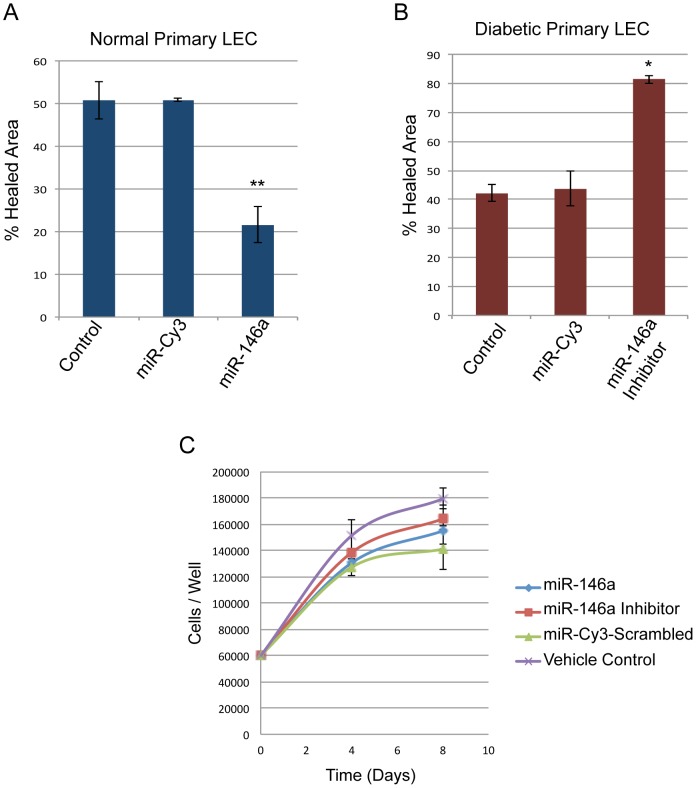
MiR-146a and wound healing quantification in primary normal and diabetic LEC. Normal and diabetic LEC were transfected with pre-miR-146a and its inhibitor, respectively, for control Cy3-labeled scrambled miRNA was used. LEC were scratch wounded, and wound closure was quantified using ImageJ software at 20 hr after wounding for normal LEC transfected with pre-miR-146a, and after 24 hr for diabetic LEC transfected with miR-146a inhibitor. **A.** Cell migration and wound closure were significantly delayed in normal primary LEC transfected with miR-146a. **B.** Conversely, wound healing was significantly enhanced in diabetic LEC transfected with miR-146a inhibitor; **C.** MTS proliferation assay showed no change in proliferation rates in primary LEC transfected with miR-146a or its inhibitor. The bar graph represents average ± SEM of pooled values three independent triplicate assays and was compared to control cells transfected with Cy3-labeled scrambled miRNA (negative control), ** p<0.009, * p<0.01 by paired two-tailed t test.

Using a telomerase-immortalized human corneal epithelial cell (HCEC) line we have previously shown that miR-146a plays a major role in the corneal epithelial wound healing [25]. In the present study, we used primary LEC from normal and diabetic corneoscleral rims to confirm our findings in the HCEC. 60–70% confluent LEC were transfected with miR-146a or its inhibitor and scratch-wounded as described [25]. To mimic a diabetic state, normal primary LEC were transfected with miR-146a. Indeed, cell migration and wound closure were significantly delayed compared to normal LEC transfected with a scrambled sequence miRNA, similar to the slow wound healing observed in diabetic corneas (Fig. 2A). In line with these results, wound healing in diabetic LEC was significantly enhanced upon transfection with miR-146a inhibitor vs. diabetic LEC transfected with scrambled sequence miRNA (Fig. 2B). To determine whether miR-146a plays a role in LEC proliferation, MTS cell proliferation assay was used. No significant change was observed in the proliferation rate of primary LEC transfected with miR-146a or its inhibitor suggesting a migratory vs. proliferative role for miR-146a in wound healing (Fig. 2C).

### Inhibition of microRNA-146a accelerates wound healing in human diabetic organ-cultured corneas

**Figure 3:**
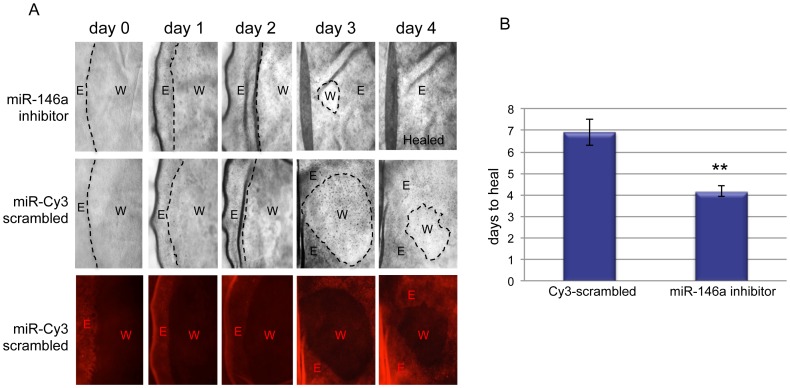
Wound healing in miR-146a inhibitor transfected diabetic organ-cultured corneas. **A.** Transfection with miR-146a inhibitor enhanced wound healing compared to control transfected with labeled scrambled miR-Cy3. Transfected diabetic organ-cultured cornea with miR-146a inhibitor, upper row; with miR-Cy3-scrambled control, middle and lower rows; fluorescence microscopy of miR-Cy3-scrambled control wound healing, lower row. E, epithelium; W, wounded area. **B.** Quantitation of wound healing rates. The bar graph represents average ± SEM of pooled values (n = 6) of days to heal. ** p<0.001 by paired two-tailed t test.

Due to epigenetic metabolic memory, organ-cultured diabetic corneas preserve protein marker alterations and display slow wound healing [29]. We attempted to normalize these alterations in human diabetic organ-cultured corneas by manipulating miRNA expression. Indeed, inhibition of miR-146a significantly enhanced cell migration vs. scrambled control in n-heptanol wounded diabetic organ-cultured corneas, with nearly a 40% decrease in average wound healing time (Fig. 3). Notably, Fig. 3 shows that wound healing rates are the greatest from day two to day three in comparison with all other time points. One possible explanation is that there is an initial latent phase during the first 24 hours of wound healing that improves significantly when cell migration approaches maximal rates somewhere between 24–36 hours depending on wound size and causative agent [9].

### Downregulation of miR-146a activates wound healing-related signaling molecules and normalizes marker expression in the diabetic cornea

**Figure 4:**
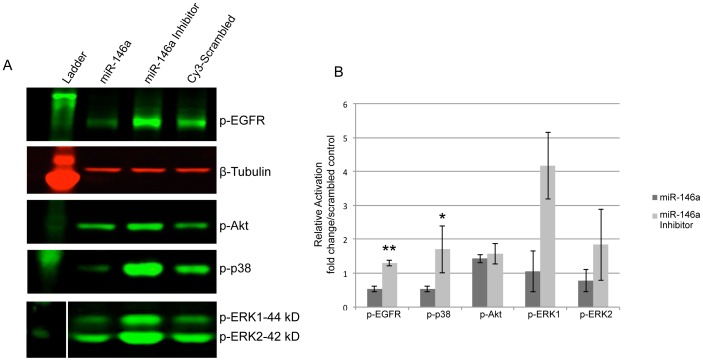
Western analyses of activated signaling molecules in transfected primary normal LEC. **A.** Total extracted protein from normal LEC transfected with pre-miRNA precursors or their inhibitors and Cy3-labeled pre-miRNA (control) was separated on gradient SDS-PAGE gels, transferred to nitrocellulose and probed with primary antibodies. Antibodies to β-tubulin or β-actin were used as loading controls and for semi-quantitation. MiR-146a treatment decreased, whereas its inhibitor increased, protein levels of p-EGFR, p-ERK1/2, p-p38, and not significantly, p-Akt. **B.** Quantitation of activated signaling molecules. The bar graph represents average ± SEM of pooled values (n = 4) of densitometric scans. *P<0.05, **P<0.01 compared with scrambled miR-Cy3 control values (taken as 1) by paired two-tailed t test.

**Figure 5:**
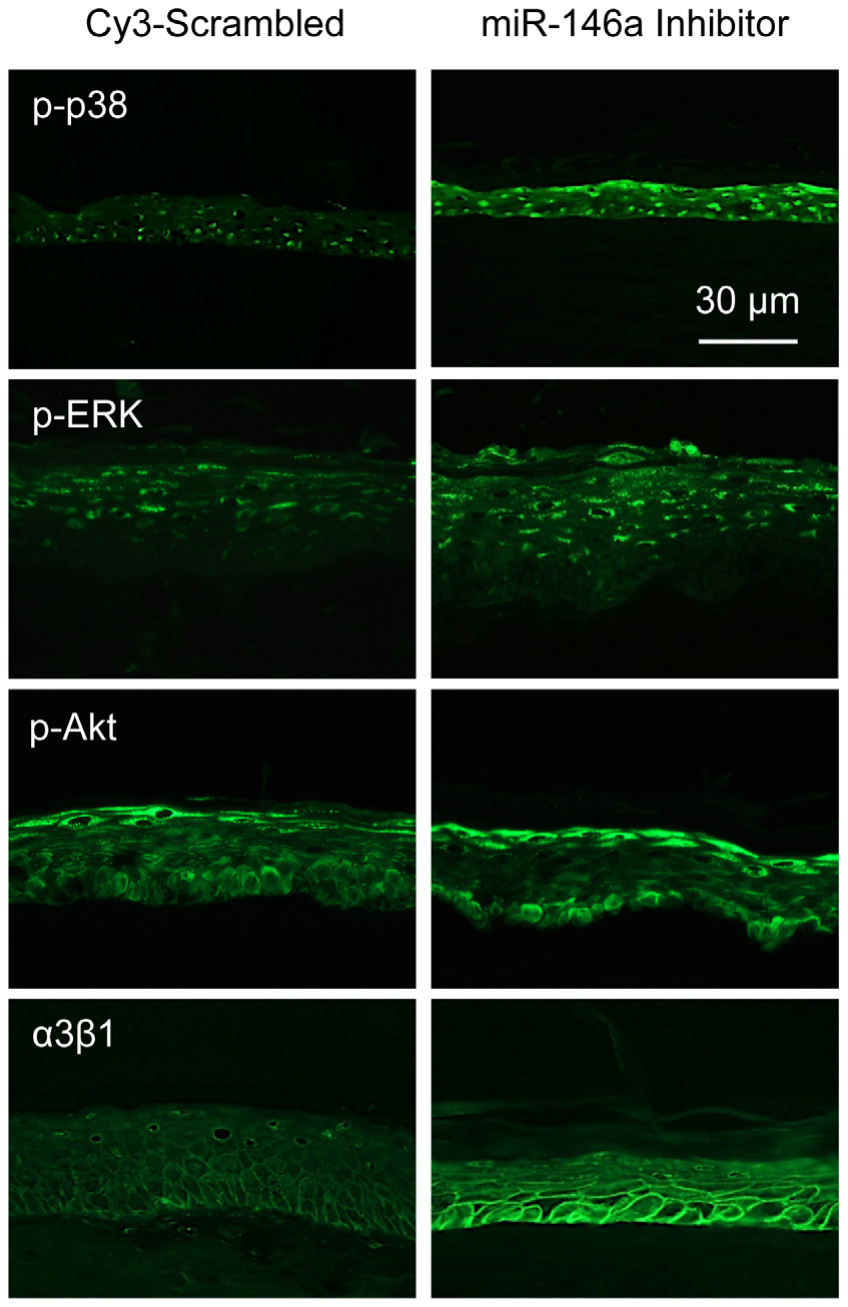
MiR-146a affects activation of wound healing-related signaling molecules and diabetic marker. MiR-146a inhibitor treatment in diabetic organ-cultured corneas led to increased expression of signaling molecules p-p38, p-ERK and p-Akt, as well as of a diabetic marker α3β1 integrin by immunofluorescent staining of limbal corneal sections. The same exposure time was used for each set of compared stained sections, and the assessment was done by more than one observer. The pictures are representative of two to three independent experiments.

We have previously shown that wounded HCEC cultures transfected with miR-146a inhibitor had elevated levels of phopsphorylated (p-) EGFR and p-p38 [25], which are important for corneal epithelial wound healing [11], [30]–[33]. Conversely, overexpression of miR-146a decreased the activation of these signaling intermediates below control levels. When primary LEC were transfected with miR-146a inhibitor, western blot analysis showed a marked increase in p-EGFR and p-p38 levels vs. the scrambled control. Additionally, some increase in p-ERK1/2 and p-Akt levels was also observed. Conversely, LEC treated with miR-146a showed a reduction in the expression of p-EGFR and p-p38 levels vs. the scrambled control, however, there was little change in p-ERK1/2 and p-Akt, possibly because endogenous miR-146a levels already ensured pronounced effect (Fig. 4). Immunostaining of organ-cultured diabetic corneas treated with miR-146a inhibitor showed a similar increase in p-p38, p-ERK and p-Akt signals (Fig. 5). Additionally, diabetic marker, integrin α3β1, markedly decreased in the diabetic cornea, returned to a normal expression pattern after inhibition of miRNA-146a in diabetic organ-cultured corneas (Fig. 5).

### Possible roles for miR-146a in LEC maintenance

**Figure 6:**
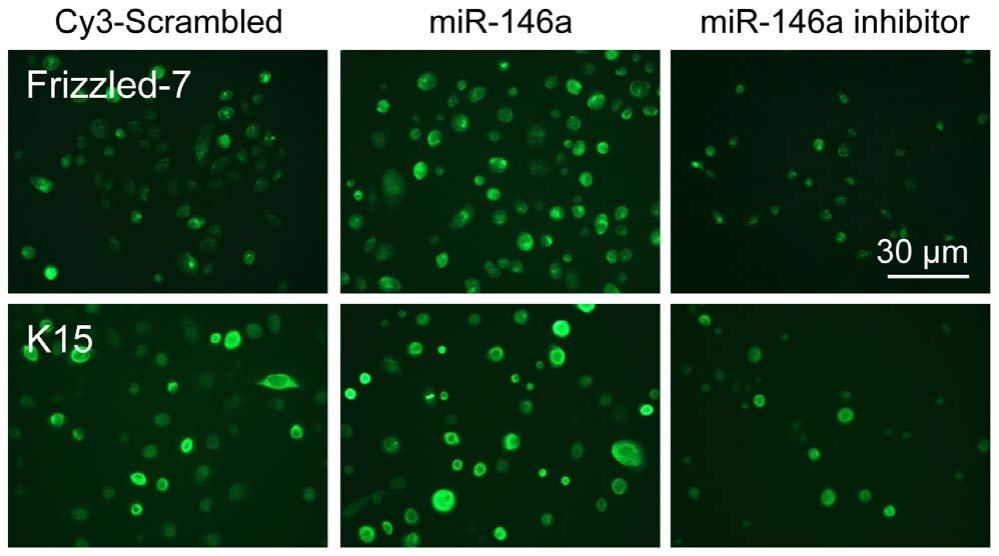
Effect of miR-146a on LESC marker expressions in diabetic organ-cultured corneas and primary LEC. **A.** Normal LEC transfected with miR-146 increased, whereas its inhibitor decreased both Frizzled-7 and K15 expression in comparison with scrambled miRNA-transfected cells. **B.** Immunofluorescent staining of limbal sections upon miR-146a inhibitor treatment of diabetic organ-cultured corneas led to decreased expression of Frizzled-7 and K15 in comparison with scrambled miRNA-transfected fellow corneas. Note that the immunostaining of the upper part of the corneal section transfected with miR-146a inhibitor is probably non-specific. The same exposure time was used for each set of compared stained sections, and the assessment was done by more than one observer. The pictures are representative of two to three independent experiments.

The effects of miR-146a and its inhibitor on the expression of putative LESC markers were also examined in normal LEC cultures and in diabetic organ-cultured corneas by immunostaining. Overexpression of miR-146a in LEC resulted in an upregulation of Frizzled-7 [34], and K15 (Fig. 6). Conversely, knockdown of miR-146a in primary LEC caused downregulation of these putative LESC markers (Fig. 6).

## Discussion

Corneal epithelium is constantly renewed by LESC that in humans and other species exclusively reside in the limbus [7]–[9], [35]. LESC have a lifetime capacity for self-renewal, and the ability to generate TA cells, which in turn differentiate into central corneal epithelial cells [9]. Deficiencies of or damage to LESC may lead to serious problems with corneal opacity and visual loss [36]–[38]. We have documented altered expression of putative LESC markers in diabetic corneas suggesting LESC dysfunction as a possible cause of diabetic keratopathy [10], [11]. The mechanisms that underlie LESC proliferation, migration, and differentiation in wound healing process could be key to understanding diabetic corneal disease. Our study is the first to employ a translational corneal gene therapy that targets disease-altered miRNAs for correction of corneal alterations, such as those caused by diabetes.

Our previous studies have identified several abnormally expressed miRNAs in diabetic corneas and their involvement in wound healing *in vitro* [25]. MiR-146a suppressed, and conversely its inhibitor enhanced, wound healing in transfected HCEC *in vitro*. Both EGFR and p-38 were activated during corneal epithelial wound healing in cells transfected with miR-146a inhibitor. Additionally, miR-146a or its inhibitor changed total EGFR in both wounded and non-wounded transfected HCEC suggesting that EGFR is one a direct targets for miR-146a [25]. Recently, miR-146a has been shown to have key roles in several diseases, such as diabetic nephropathy-associated inflammation [39], Graves' ophthalmopathy [40] and diabetic retinopathy [41], [42]. MiR-146a polymorphisms have been associated with leprosy in neural cells [43], non-small cell lung cancer [44], and hepatocellular carcinoma [45], [46]. These data suggest that miR-146a is an important regulator in many normal and disease processes.

MiR-146a has been shown to be an important regulator of a wide variety of cell functions, and is able to target different genes in different cell types, which has led to some controversy in regard to its role both as tumor suppressor and oncogene in different types of cancers [47]–[49]. Increasing evidence suggests that miR-146a is involved in the innate immune and inflammatory response, and tumorigenesis [39], [41], [47]–[49]. However, its role and mechanism of action remains to be fully elucidated in normal and pathological conditions. These roles must be understood in order to use it as a therapeutic agent. MiR-146a has a growing list of presumed targets including, TRAF6, IRAK1, CXCR4, NF-κB, which may contribute to inflammation and tumor development [50]–[55]. MiR-146a association with Smad4 mRNA and TGF-β pathway components suggests an involvement in cell proliferation [56], whereas alteration of EGFR and its downstream signaling molecules via miR-146a expression demonstrates an inhibitory effect on cell migration and invasion [25], [48].

Migration and proliferation are essential components of corneal epithelial homeostasis suggesting that miR-146a has a negative regulatory role in corneal epithelial wound healing.

Wound healing is a complex multistep process that involves consecutive changes in many genes including those that encode miRNAs in corneal epithelial cells. Since miRNAs contribute to regulating and balancing gene expression networks to maintain corneal homeostasis, their dysregulation potentially tips this dynamic process contributing to pathological conditions such as diabetic keratopathy.

In the present study, we confirmed that miR-146a expression was also altered in the diabetic vs. normal limbus. Its upregulation in the limbus vs. central cornea was also confirmed by QPCR in addition to in situ hybridization [25] suggesting its possible role in limbal epithelial cell homeostasis. We then tested the effects of miR-146a in wound healing of primary LEC and organ-cultured diabetic corneas. We aimed at unraveling the functional importance of miR-146a in diabetes-associated corneal changes and underlying molecular mechanisms, to further understand its roles in corneal homeostasis. Overexpression of miR-146a in normal LEC delayed wound healing vs. normal LEC transfected with scrambled sequence miRNA, whereas wound healing was enhanced in diabetic LEC transfected with miR-146a inhibitor vs. diabetic LEC transfected with scrambled sequence control miRNA. Furthermore, human organ-cultured diabetic corneas transfected with miR-146a inhibitor showed acceleration of corneal epithelial wound healing impaired in diabetes, and normalized patterns of a diabetes-associated corneal marker, α3β1 integrin [57]. α3β1 integrin is a member of integrin family that serves as a receptor for several extracellular matrix components with a complex role in modulating adhesion, migration and cytoskeletal organization [58], [59]. Since it regulates adhesion and migration during wound healing, thereby its downregulation in diabetic corneal epithelium could be one of the causes contributing to impairment in wound healing in these tissues. It may thus be suggested that miR-146a contributes to the clinically observed abnormalities seen in corneas of diabetic patients.

It is well documented that p-EGFR signaling and its downstream effectors, p-p38 (MAPK) and p-Akt are directly involved in corneal epithelial wound healing in diabetic cornea, whereas, their total protein contents did not differ significantly [30]–[33]. Consistent with our previous findings [25], we showed that inhibition of miR-146a enhanced corneal epithelial wound healing in primary LEC and diabetic organ-cultured corneas via EGFR signaling and subsequent activation of the ERK - p38 and to a lesser extent, PI-3 kinase - Akt pathways. By western analysis, p-EGFR and p-p38 were reproducibly upregulated in primary LEC transfected with miR-146a inhibitor. Organ-cultured diabetic corneas transfected with miR-146a inhibitor also showed upregulation of activated p38 and ERK by immunohistochemistry. In agreement with these data, p-EGFR and p-p38 were downregulated upon overexpression of miR-146a in primary normal LEC. Although there was some change in the expression levels of p-ERK1/2 and p-Akt, it did not reach significance using both western blot and semi-quantitative immunostaining, possibly because endogenous miR-146a levels already ensured pronounced effect. These data are in good agreement with our previous study using HCEC line [25]. Therefore, upregulation of miR-146a, which decreases the expression and transactivation of its direct target, EGFR, may be responsible for the slow wound healing in diabetic organ-cultured corneas but may not be directly involved in wound healing process per se.

Other recent studies have also shown that miR-146a negatively regulates EGFR expression and inhibits tumor growth through the MAPK kinase pathway in a p-ERK-dependent manner to control cell migration, proliferation, and morphogenesis. [47], [48]. EGFR is a powerful mediator of corneal epithelial wound healing and can signal through Src - PI-3 kinase - Akt survival axis, or through Ras – ERK - MAPK. Our previous data using *c-met* (hepatocyte growth factor receptor) gene therapy in human organ-cultured diabetic corneas showed that accelerated epithelial wound healing was dependent on p38, but not on EGFR - Akt activation [31]. At the same time, this acceleration, when achieved through silencing of the proteinase *MMP-10* and *cathepsin F* genes, was apparently due to activation of the EGFR - Akt axis, but not p38 [11]. The present results suggest that EGFR, a miR-146a target, may effect corneal epithelial wound healing through the Ras – ERK - p38 cascade. Our data also suggest that miR-146a is associated with undifferentiated LEC at the corneal periphery, and its expression is downregulated in the central cornea populated by differentiated cells. Therefore, we speculate that miR-146a may play a role in maintenance and/or early differentiation of LESC and TA cells due to its upregulation in limbus vs. central cornea. This is supported by upregulation of putative LESC markers, K15 and Frizzled-7, in miR-146a-transfected normal LEC. This notion should be addressed in future studies. Furthermore, abnormal miR-146a upregulation may be an important mechanism of delayed wound healing in the diabetic cornea.

In summary, inhibition of diabetes-elevated miR-146a in organ-cultured diabetic corneas normalized epithelial wound healing and activated signaling molecules, EGFR and p38. Our miRNA gene therapy using miR-146a inhibitor significantly enhanced wound healing rates and the expression of specific corneal epithelial markers. These data attest to the significance of miRNAs in regulating corneal cell homeostasis, and provide evidence that manipulating their expression levels has a potentially high impact for alleviating disease symptoms.
